# Primary surgical bleeding and platelet function are unchanged in heartworm-infected dogs

**DOI:** 10.3389/fvets.2026.1773626

**Published:** 2026-05-07

**Authors:** Peyton A. Cole, Carisa Fraser, Mandy L. Wallace, Hannah Jordan, Amie Koenig, Benjamin M. Brainard

**Affiliations:** 1 Department of Small Animal Medicine & Surgery, College of Veterinary Medicine, University of Georgia, Athens, Georgia; 2 Department of Pathology, College of Veterinary Medicine, University of Georgia, Athens, Georgia

**Keywords:** canine, coagulation, dirofilaria immitis, platelet, thromboelastography

## Abstract

**Introduction:**

Heartworm-infected dogs may have changes in platelet function which can manifest as either perioperative bleeding or thrombosis, reflecting either a prothrombotic state from platelet hyperreactivity or hypocoagulability secondary to consumptive coagulopathy. We aimed to evaluate surgical bleeding in heartworm-infected dogs undergoing ovariohysterectomy (OHE).

**Methods:**

Heartworm-infected (HWI, *n* = 11) and uninfected (UI, *n* = 21) dogs undergoing elective OHE were included. Dogs were anesthetized using a standard protocol, and platelet and coagulation function was evaluated using buccal mucosal bleeding time (BMBT), platelet function analyzer-100 (PFA-100), and thromboelastography (TEG). Bleeding from the initial skin incision was quantified using pre-weighed sponges. Variables between HWI and UI dogs were compared using *t*-tests or Mann-Whitney Rank Sum Tests as appropriate for data distribution.

**Results:**

Blood loss measured by surgical sponge weight did not differ between groups: median (25–75th percentile) was 7 g (6–10 g) in HWI dogs and 7 g (6.3–9.8 g) in UI dogs (*P* = 0.490). PFA-100 closure times were 79.6 ± 15 s in HWI dogs and 83.7 ± 22 s in UI dogs (*P* = 0.571). Similarly, no significant differences were identified between groups for CBC or TEG measurements or BMBT.

**Discussion:**

HWI dogs did not experience increased incisional bleeding or quantifiable differences in platelet and coagulation function tests compared to UI dogs. HWI dogs undergoing ovariohysterectomy in this study did not display tendencies for perioperative hemorrhage or thrombosis.

## Introduction

Heartworm (HW) disease resulting from infection with *Dirofilaria immitis* affects dogs throughout the United States. Within the US, HW disease is present in all states and territories, and is also an important canine disease in Europe, Asia, South America, and Africa (1). Infection with *D. immitis* can be fatal when left untreated, resulting in pulmonary hypertension and right-sided heart failure, and may progress to caval syndrome, which is characterized by pulmonary outflow obstruction and circulatory shock (2). The mechanisms underlying HW infection-induced vascular pathology are not completely understood but can be characterized by worm emboli with evidence of clot formation around the embolus (3). Some reports have demonstrated increased platelet reactivity in dogs experimentally infected with *D. immitis*, suggesting increased platelet function and a prothrombotic or hypercoagulable state, (4, 5) but a recent report evaluating platelet function under high shear conditions (to simulate *in vivo* blood flow) did not identify enhanced platelet activity in HW positive dogs (6). Another report evaluating viscoelastic coagulation testing in naturally-infected HW positive dogs described slight, relative hypercoagulability (7). Taken together, the evidence suggests that dogs with HW infection may present with either a hypercoagulable state that predisposes to development of thrombosis or a hypocoagulable state due to consumption of platelets and clotting factors following (thrombo)embolic disease.

Understanding the effects of *D. immitis* infection on coagulation function is especially important considering that HW disease disproportionately affects the shelter dog population, (6) where sterilization procedures are commonly performed prior to the adoption process. Previous studies did not document an increase in perioperative complications in heartworm infected (HWI) dogs in a shelter population undergoing sterilization (8). Current recommendations from the American Heartworm Society support the performance of sterilization procedures in HW positive dogs with absent to mild clinical signs of HW disease. Although the ovariohysterectomy procedure is considered relatively safe, surgery could have serious consequences in patients with unidentified coagulation dysfunction. Therefore, it is important to know if HWI dogs are at an increased risk for bleeding or thrombosis when these surgeries are being performed in low resource or shelter environments with limited access to blood products and intensive care.

The purpose of this study was to determine if platelet function and global coagulation function was altered in dogs with naturally occurring HW infection undergoing elective ovariohysterectomy (OVH). Platelet function was evaluated using a platelet function analyzer-100 (PFA-100) and buccal mucosal bleeding time (BMBT), in addition to a CBC with blood smear to verify platelet count and microfilaremia. Global coagulation activity was evaluated using thromboelastography (TEG). Incisional bleeding, thought to be representative primarily of platelet function, was quantified using blood loss absorbed by pre-weighed surgical sponges during the abdominal midline incisions in dogs undergoing OVH. Any surgical or coagulation related complications in the 48 h. following surgery were evaluated through daily postoperative physical examinations.

## Materials and methods

### Study population

The study population was recruited from female, presumably intact dogs residing at four animal shelters across the state of Georgia, USA, that were to be sterilized as part of a veterinary student teaching laboratory at the University of Georgia College of Veterinary Medicine in the year 2020. Dogs were considered for inclusion if they weighed over 10 kg and were at least 6 months of age. If no age was reported by the shelter or only an estimated age was available, dentition was evaluated to confirm that the dog was greater than 6 months old. Physical examinations were performed by veterinary students and supervised by licensed veterinarians. Physical exam included cardiorespiratory assessments including cardiac and pulmonary auscultation, assessment of mucous membrane color and capillary refill time, and palpation of pulses for relative strength and synchrony with the heart beat. Heartworm infection status was confirmed by blood film evaluation for identification of circulating microfilaria and a lateral flow HW antigen test (4Dx SNAP test, IDEXX). Dogs were considered to be HWI if they were HW antigen positive with or without circulating microfilaria observed on blood smear evaluation. Dogs that were antigen negative were considered UI. This study was approved by the University of Georgia's IACUC (AUP# A2018 06-004-A7), and approval was granted by all participating animal shelters.

### Anesthetic protocol

All dogs were premedicated with acepromazine (0.02 mg/kg) and hydromorphone (0.1 mg/kg) given intramuscularly. 20 min later they were induced to anesthesia with propofol (4.4 mg/kg, IV) to facilitate intubation. Isoflurane delivered in 100% oxygen was utilized to maintain an appropriate anesthetic plane during surgery, and carprofen (2.2 mg/kg, SQ) was administered for post-operative analgesia.

### Blood sampling and clinicopathological testing

All dogs were under general anesthesia prior to blood sample collection. 7 ml of whole blood was acquired via jugular venipuncture using a 20-ga needle attached to a 6 ml syringe. Following venipuncture, 4.5 ml of whole blood was divided between two tubes containing 3.2% citrate to obtain a citrate: blood ratio of 1:9. The remainder of the blood was placed into tubes containing EDTA. The EDTA-anticoagulated blood was used to generate a CBC including platelet concentration estimates (ADVIA 2120i, Siemens HealthCare Diagnostics, Deerfield, IL). A fresh blood smear was also made, which was reviewed by a medical technologist and one investigator (CF) blinded to the heartworm status of the dog. Following this analysis, the samples were centrifuged and plasma decanted and stored at −80 °C prior to HW antigen testing using a commercial lateral flow assay (SNAP 4Dx, IDEXX Laboratories, Inc., Westbrook, ME). All coagulation testing was performed within 1 h of collection by two investigators (BMB and CF). Although attempts were made to analyze the TEG samples following a 30 min rest period, transport logistics resulted in some delays (no more than 1 h from collection) in the initiation of TEG analysis.

Platelet function under high-shear conditions was evaluated using a commercial analyzer (PFA-100, Siemens HealthCare Diagnostics, Deerfield, IL) and cartridges with an aperture lined with collagen and ADP (COL/ADP). 800 μl of citrated whole blood was pipetted into the PFA cartridge reservoir, and the test was run until the machine indicated an occlusion to the aperture (closure time [CT]). Assays were performed once per individual or duplicated if CT exceeded 300 s or if an error was reported.

Thromboelastography was performed using a commercial viscoelastic coagulation analyzer (TEG 5000 Hemostasis Analyzer, Haemonetics, Boston MA) using recalcified, citrated whole blood activated by recombinant human tissue factor (TF, Dade Innovin, Siemens Healthcare Diagnostics, Tarrytown, NY) in a final concentration of 1:3,600. This methodology has been previously described for use in evaluation of canine blood (6). The following TEG parameters were recorded: reaction time (R), clotting time (K), angle (α), maximal amplitude (MA), and global clot strength (G).

### Buccal mucosal bleeding time

Buccal Mucosal Bleeding Time was performed using standard techniques (9). Briefly, while patients were under general anesthesia, a commercial lancet (Surgicutt; International Technidyne Corporation, Piscataway, New Jersey) was used to make a standard incision into the buccal mucosa by a single operator (CF). Once the lancet was discharged, filter paper was used to gently wick away pooled blood, taking care to not disturb the forming thrombus. Time until clot formation was measured by a dedicated stopwatch.

### Incision length and sponge weight

All dogs undergoing OVH surgery had abdominal fur clipped, and the surgical site sterilely prepared. As previous studies raised concern for changes to platelet function, we focused on clinical assessment of platelet activity for this study by measuring blood loss from the initial skin incision. One sterilized pack including pre-weighed gauze sponges and a disposable centimeter ruler were dispensed to each surgery group. The pre-weighed sponges were used to achieve primary hemostasis by the surgeon prior to incision of the linea alba. If primary hemostasis could not be achieved with repeated compression using the sterile gauze, hemostats were employed. All used sponges were sterilely collected by a research assistant. The pre-weighed gauze sponges were removed from the surgical field and weighed to quantify the amount of incisional blood loss per patient. The student groups used the sterilized paper ruler to measure the incision length in centimeters. Additional recorded parameters included the time of initial incision and the time at which the surgeon entered the abdominal cavity, after which the surgery and postoperative care proceeded routinely. No dogs experienced intraoperative hemorrhage.

### Statistical analyses

Based on prior research, our goal was to recruit at least 10 dogs for each group, with a goal of detecting a difference of 3 g. of sponge weight between HWI and UI dogs (6). Statistical analyses were performed using commercially available software (GraphPad Prism and Sigmaplot 14: systat inc.). Data were assessed for normality and variance using Shapiro-Wilk and Brown-Forsythe tests, respectively. Continuous variables between HWI and non-infected dogs were compared using unpaired, two-tailed *t*-tests or Mann-Whitney Rank Sum Tests as appropriate for the distribution of the data. Relationships between body weight and surgical sponge weight and the incision length were accomplished with Pearson correlation analysis. The alpha value was set at 0.05.

## Results

The study population consisted of 32 female, presumably intact dogs. The mean age was 3.5 years in dogs in the HWI group (*n* = 11) and 1.7 years in the uninfected group (*n* = 21) (*P* = 0.035). Mean weight of the HWI dogs was 23.87 kg, while that in the uninfected group was 19.40 kg (*P* = 0.0597). Body condition scoring (BCS) was performed using a 9 point scale, (10) and all dogs but two were judged to have a BCS of either 4 or 5/9. The remaining dogs were recorded as a BCS of 7/9, indicating moderate obesity. Surgical data from one dog was not included in blood loss analysis, as a spay scar was identified on the abdomen during surgical site preparation. This dog was included in coagulation testing, as blood was drawn prior to identification of the scar, and these data were included in the overall analyses. Major comorbidities were not identified in any of the recruited dogs. Overall, 11 dogs were considered to be HWI, 10 of which were microfilaria positive. 15 dogs (47%) were infested with one or more of fleas, ticks, hookworms, and/or whipworms. Five of these dogs were in the HWI group.

Monocyte counts were significantly lower in the HWI group (*P* = 0.0413), but other red and white blood cell indices, including hematocrit, were not different between groups. Platelet number, plateletcrit (PCT), mean platelet volume (MPV), platelet distribution width (PDW), and platelet reticulocyte count did not significantly differ between groups. All median values were within institutional reference intervals (see Supplementary Table 1).

**Figure 1:**
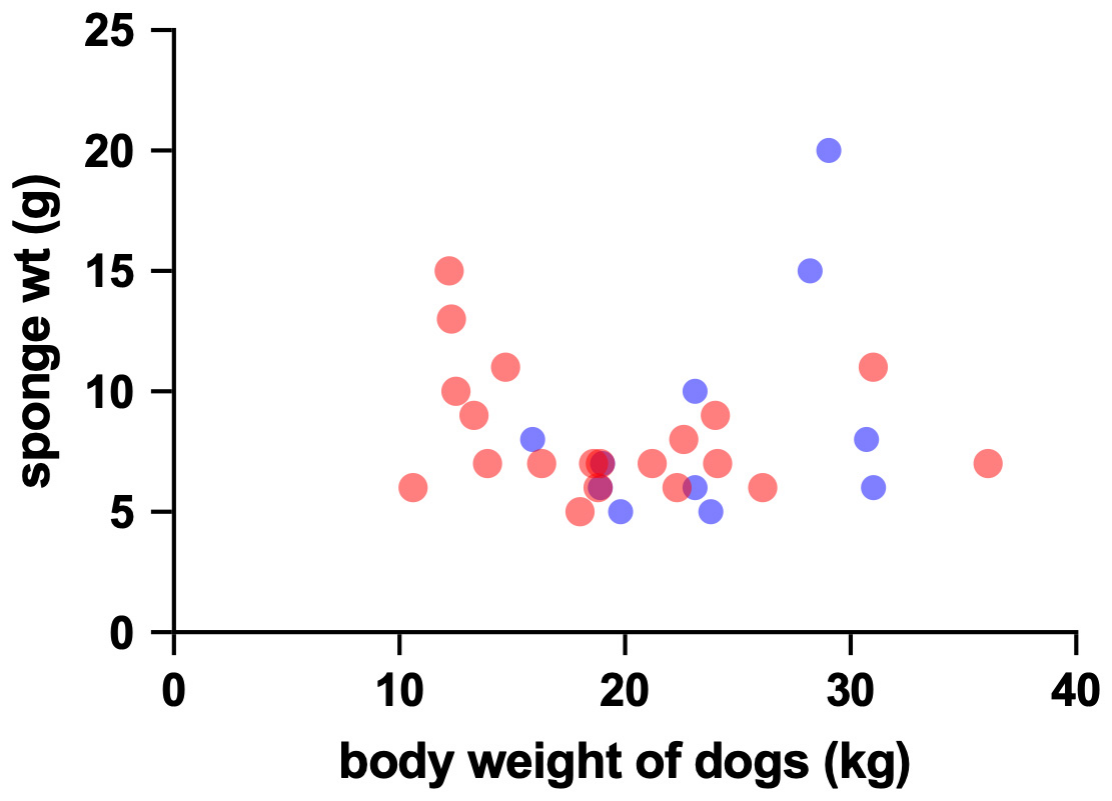
Graphical display of the association between body weight of dogs and the sponge weight after skin incision (sponges weighed 5 g prior to surgery). Heartworm positive dogs are depicted by blue dots, and uninfected dogs by red dots.

**Figure 2:**
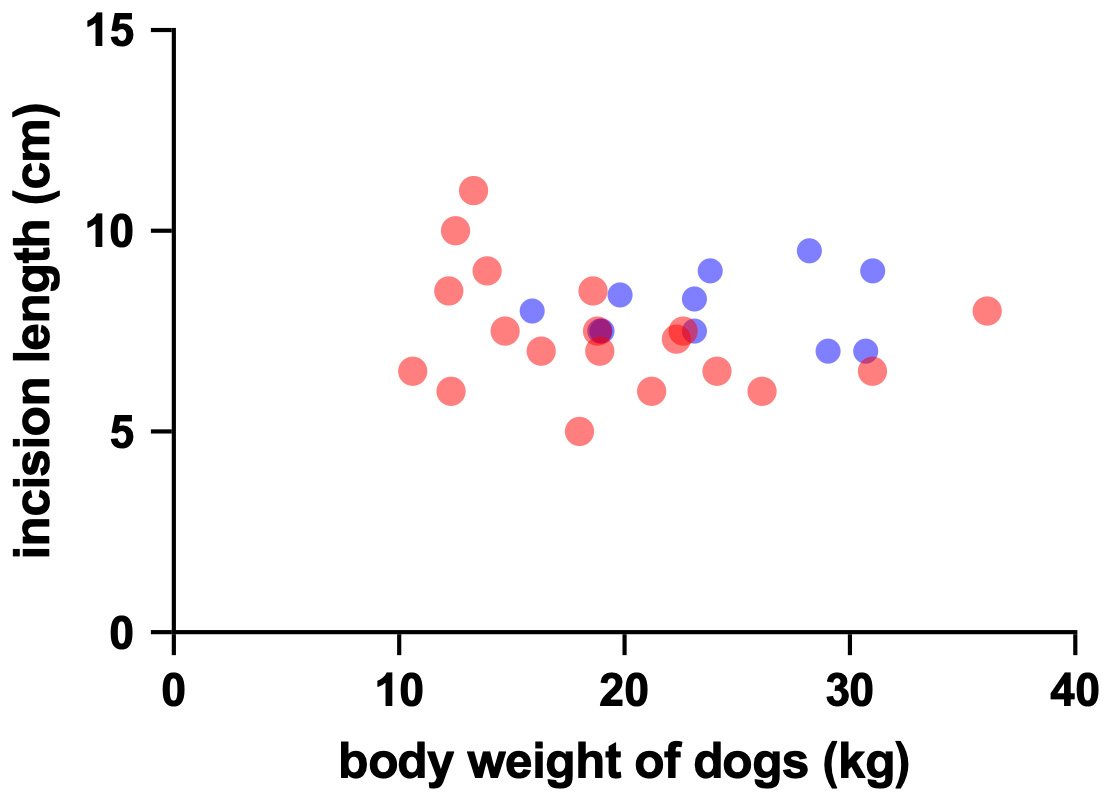
Graphical display of the association between body weight of dogs and the length of skin incision. Heartworm positive dogs are depicted by blue dots, and uninfected dogs by red dots.

Closure time measured by PFA-100 was not significantly different between groups, and the TEG parameters R, K, α, MA, and G did not differ between groups (Table 1). BMBT was 138.1 +/−55 s in the HWI group and 172.8 +/−58 s in the uninfected group (*P* = 0.112). Skin incision length was 8.070 +/−0.9 cm in the HWI group and 7.437 +/−1.5 cm in the uninfected group (*P* = 0.228). Median surgical sponge weight following use was 7 g (6–10 g, 25–75th percentile) in the HWI group and 7 g (6.25 g−9.75 g, 25th−75th percentile) in uninfected dogs (*P* = 0.490). The relationship of dog body weight to sponge weight after use and to incision length are graphed in Figures 1, 2, respectively. There was also no relationship between body weight and sponge weight (*P* = 0.789) or incision length (*P* = 0.584).

**Table 1:** Selected TEG and PFA-100 results were not significantly different in HWI and uninfected dogs.

Parameter	Reference interval	Group	Mean	Std dev	*P*-value
R-time (minutes)	1.6–2.4	HWI	2.1	0.29	0.769
		Uninfected	2.2	0.38	
K-time (minutes)	2–4	HWI	1.2	0.35	0.281
		Uninfected	1.1	0.24	
α-angle (degrees)	42–68	HWI	72.8	4.10	0.296
		Uninfected	74.7	3.50	
MA (degrees)	45.6–63.2	HWI	58.7	7.35	0.367
		Uninfected	61.5	5.32	
G (kdyne/sec)	4–8	HWI	7.4	1.99	0.375
		Uninfected	8.2	2.08	
PFA-100 CT (seconds)	52–86	HWI	79.4	15.11	0.571
		Uninfected	83.7	22.20	

## Discussion

This study aimed to evaluate whether a population of dogs naturally infected with *D. immitis* demonstrated altered platelet or coagulation function. No significant differences in platelet function as evaluated by PFA-100, BMBT, and CBC were identified between HWI and uninfected dogs. TEG was performed to evaluate global coagulation competence and showed no significant differences in clotting time (R and K), clot formation rate (α), or clot strength (MA and G). Perioperative incisional bleeding during elective OVH as evaluated through surgical sponge weight also did not significantly differ between groups. There were no observed or reported complications of the surgeries that could be ascribed to either thrombosis or bleeding. Therefore, this study failed to demonstrate evidence of either a hypocoagulable or hypercoagulable state associated with naturally occurring HWI. These results suggest that asymptomatic HWI does not increase the risk of hemorrhage in this population of dogs undergoing elective abdominal surgical procedures. Taken along with other reports, (7) in dogs without clinical signs of heartworm disease, sterilization and other elective procedures may be considered safe without increased risk of perioperative coagulation complications.

The decision to include PFA, BMBT, and sponge-weight in addition to platelet indices in this study was to allow us to focus specifically on platelet function in HWI dogs (4, 5). Although BMBT is a more clinically available and inexpensive way to evaluate platelet function than the PFA, it cannot be relied on as a sole assay for assessment because it is not sensitive enough to identify subtle primary hemostatic changes that might impact surgical bleeding but which do not exceed the (relatively wide) BMBT reference interval. A prior canine study (11) showed a fair correlation (*r* = 0.56) of BMBT with intraoperative blood loss, but while two dogs with prolonged BMBT experienced increased intraoperative blood loss, there were also two dogs with greater than expected intraoperative blood loss that had BMBT within the reference interval. The sensitivity of template bleeding time tests for detecting primary hemostatic disorders in humans is reported to be between 50 and 65% (12). From this perspective, a very prolonged BMBT (e.g., lasting longer than 6 min) likely indicates a significant platelet defect (and so may be useful to a practitioner working on a dog with a high suspicion for platelet dysfunction like vonWillebrand's disease) but based on our study, we cannot recommend reliance on BMBT alone to determine whether or not platelet function is ‘normal' to the degree where it will not impact surgical bleeding.

TEG was chosen for evaluation of animals in this study based on the results of a prior report which identified an increase in angle and MA in HWI dogs (6). These findings would theoretically be consistent with hypercoagulability, and in that report were thought to be associated with a significantly higher fibrinogen concentration in the HWI dogs (6). Hypercoagulability as determined by viscoelastic coagulation testing using a different analyzer was also suggested in another recent report, although values in both tested groups were within the provided reference intervals of that analyzer (7). TEG would also have identified animals with hypocoagulability, and so may be a useful screening test in this context.

All dogs included in the report from Fraser et al. had a known duration of HW infection of at least 1 year (6). In the current study and in that of Newmans et al., (7) the duration and severity of infection prior to arrival at the shelter was unknown. There was also no attempt to further quantify the severity of heartworm disease in the dogs of this report based on additional diagnostics or follow-up after treatment of heartworm disease. Young animals undergoing earlier spay and neuter procedures likely have a lower chance of harboring an advanced HW infection, considering it takes at least 6 months to develop a patent infection following inoculation, (2) but we also did not know the duration of HWI in the infected dogs (i.e. the age may be irrelevant if the infection was relatively recent, in the absence of other inflammatory disease).

It is not clear if duration of infection may influence overall coagulation profile, but sustained inflammation due to prolonged HW infection may increase fibrinogen concentrations, resulting in viscoelastic coagulation profiles consistent with hypercoagulability. We cannot definitively predict the risk based on the duration of HW infection, but HWI dogs with a shorter course of infection may have lower degrees of inflammation, which may be translated into TEG results that do not suggest hypercoagulability. Interestingly, a recent study found elevations in C-reactive protein (a marker of systemic inflammation) in HWI dogs was more associated with pulmonary arterial changes, rather than just worm burden, which also associates inflammation with the duration of infection (13). Thoracic radiography or echocardiography may have also been helpful in this study to estimate duration of HWI through identification of changes consistent with chronic HWI (e.g., pulmonary arterial enlargement, pulmonary parenchymal lesions) (14).

Despite the HWI animals being older, we did not identify differences in coagulation that might be ascribed to systemic inflammation (from HWI or other inflammatory disease like osteoarthritis). We did not, however, perform more specific assays to gauge systemic inflammation (e.g., fibrinogen or c-reactive protein concentrations), which may have provided us with an alternative categorization to analyze the collected data, and may be useful analytes for future studies. In addition, gastrointestinal parasitism and dermatologic conditions present in both groups in our study may have contributed to inflammation and mitigated differences in inflammation that could be solely attributable to HWI between groups.

The anesthetic protocol used in this study utilized acepromazine, which has been suggested to cause platelet dysfunction (15). We did not identify alteration of platelet function using multiple testing modalities including BMBT, PFA-100, and TEG. This is also consistent with a more recent study that did not identify platelet function inhibition following acepromazine administration to dogs at greater dosages than were used in this study (16).

There are several limitations of this study. First, although anesthetic protocols were standardized and incision length did not statistically vary between groups, a different surgeon performed each individual ovariohysterectomy. The surgeries were performed by veterinary students with limited surgical experience, unlike surgeries that are performed in a high volume shelter setting, as reported by Newmans et al. (7). This indicates that the risk of bleeding in HWI dogs is unlikely to be impacted by surgical skill or timing, and provides some buffer for animals undergoing longer procedures. Another limitation of the current study is that blood loss was only measured during the initial skin incision through sponge weight. This may have been inadequately sensitive to document blood loss, as it did not quantify blood lost during the entire perioperative period (although we can verify that bleeding severe enough to cause hemoperitoneum or clinical hypovolemia was not identified in the 48 h after surgery). The number of dogs with a positive heartworm test was small and the severity of heartworm infection was not assessed, although none of the dogs were symptomatic. The number of dogs in each group (HWI vs. uninfected) could not be planned prior to arrival of the dogs at the teaching hospital; however, based on a prior study (6) and initial investigation of sponge weights (and verified by the standard deviation of the sponge weights measured in this study), the identification of a 3 g greater sponge weight in the HWI group would require a minimum of eight dogs in each group for adequate power. Although the dogs were lost to long term follow up, no complications were observed in the immediate 48 h perioperative period. There is no way to evaluate if any HWI dogs developed thrombosis after this observation period.

This study did not identify differences in platelet function, coagulation competence, or incisional blood loss between HWI or uninfected shelter dogs undergoing elective ovariohysterectomy. This suggests that ovariohysterectomy can be performed safely in otherwise healthy HWI dogs without an increased risk of blood loss related to decreased platelet function, at least during the initial approach to the abdomen. The presence of HWI should not preclude the decision to perform ovariohysterectomy in asymptomatic patients.

## Data Availability

The raw data supporting the conclusions of this article will be made available by the authors, without undue reservation.
