# Supplementary material for: Primary surgical bleeding and platelet function are unchanged in heartworm-infected dogs
Source: Front Vet Sci. 2026 May 7;13:1773626. doi: 10.3389/fvets.2026.1773626 (PMC13189967; doi:10.3389/fvets.2026.1773626)
Supplement: Supplementary file 1 [file Table_1.docx]

**Supplemental Table 1**. Selected WBC, RBC, and platelet parameters in dogs with heartworm infection (HWI, n=11) and uninfected dogs (n=21).

| **Parameter** | **Reference Interval** | **Group** | **Mean** | **Std Dev** | **P Value** |
| --- | --- | --- | --- | --- | --- |
| HCT (%) | 42.2 - 59.8 | HWI  Uninfected | 39.791  37.852 | 3.289  3.682 | 0.143 |
| WBC (x 10^3/ul) | 4.2 - 12.9 | HWI  Uninfected | 8.873  8.788 | 1.861  3.475 | 0.592 |
| Segmented  Neutrophils  (x 10^3/ul) | 2.700 - 8.500 | HWI  Uninfected | 5.768  5.719 | 1.790  2.417 | 0.843 |
| Band  Neutrophils  (x 10^3/ul) | 0.000 - 0.3000 | HWI  Uninfected | 0.00536  0.00605 | 0.0178  0.0277 | 0.705 |
| Lymphocytes  (x 10^3/ul) | 0.500 - 4.100 | HWI  Uninfected | 1.716  1.838 | 0.753  0.188 | 0.984 |
| Monocytes  (x 10^3/ul) | 0.100 - 1.000 | HWI  Uninfected | 0.225  0.373 | 0.181  0.188 | 0.043 |
| Eosinophils  (x 10^3/ul) | 0.000 - 1.200 | HWI  Uninfected | 1.158  0.848 | 0.603  0.595 | 0.165 |
| Basophils  (x 10^3/ul) | 0.000 - 0.045 | HWI  Uninfected | 0.000  0.00281 | 0.000  0.0129 | 0.511 |
| Abs Reticulocytes  (x 10^9/L) | 0.0 - 91.0 | HWI  Uninfected | 33.217  36.771 | 11.062  13.394 | 0.431 |
| Platelets  (x 10^3/ul) | 226 - 424 | HWI  Uninfected | 339.000  300.810 | 63.662  72.116 | 0.138 |
| MPV (fl) | 8.9 - 14.4 | HWI  Uninfected | 9.664  10.424 | 2.052  1.604 | 0.300 |
| PDW (%) | 39.9 - 67.2 | HWI  Uninfected | 63.836  65.300 | 9.301  4.558 | 0.631 |
| PCT (%) | 0.15 - 0.45 | HWI  Uninfected | 0.327  0.310 | 0.0980  0.0755 | 0.591 |
| MPC (g/dl) | 17.2 - 23.6 | HWI  Uninfected | 23.755  23.767 | 1.440  1.436 | 0.889 |
| %RtcPlts (%) | unspecified | HWI  Uninfected | 1.343  1.672 | 1.029  1.130 | 0.117 |
| RtcPlts Count  (x 10^3/ul) | unspecified | HWI  Uninfected | 32.364  38.714 | 18.184  15.850 | 0.340 |
